# Prostate cancer survivors: Risk and mortality in second primary cancers

**DOI:** 10.1002/cam4.1764

**Published:** 2018-10-01

**Authors:** Subhayan Chattopadhyay, Guoqiao Zheng, Otto Hemminki, Asta Försti, Kristina Sundquist, Kari Hemminki

**Affiliations:** ^1^ Division of Molecular Genetic Epidemiology German Cancer Research Center (DKFZ) Heidelberg Germany; ^2^ Faculty of Medicine University of Heidelberg Heidelberg Germany; ^3^ Department of Abdominal Surgery and Urology Helsinki University Hospital Helsinki Finland; ^4^ Cancer Gene Therapy Group Faculty of Medicine University of Helsinki Helsinki Finland; ^5^ Center for Primary Health Care Research Lund University Malmö Sweden; ^6^ Department of Family Medicine and Community Health Department of Population Health Science and Policy Icahn School of Medicine at Mount Sinai New York New York; ^7^ Center for Community‐based Healthcare Research and Education (CoHRE) Department of Functional Pathology School of Medicine Shimane University Matsue Japan

**Keywords:** incidence, mortality, multiple primary cancers, relative risk, screening

## Abstract

To assess etiological and clinical consequences of second primary cancers (SPCs) in prostate cancer (PC) patients, we followed newly diagnosed patients to identify men who were diagnosed with a SPC and recorded their causes of death. We used the Swedish Family‐Cancer Database to assess relative risks (RRs) and causes of death in SPCs until the year 2015 in patients with a PC diagnosis between 2001 and 2010. Among a total of 4.26 million men, 76 614 were diagnosed with PC at the median age of 71 years. Among them, 8659 (11.3%) received a subsequent diagnosis of SPC after a median follow‐up of 4 years. The most common SPCs were colorectal, skin, bladder, and lung cancers, melanoma, and non‐Hodgkin lymphoma. The ranking was almost identical with first cancers among elderly men in Sweden. The RR for SPCs in prostate‐specific antigen—detected PC was approximately equal to RR in other PC. Mortality patterns of PC patients were distinct depending on the presence or absence of SPC. Among patients with SPC, 47.8% died as a result of the corresponding SPC, followed by other causes (22.2%) and PC (18.1%). For patients without SPC, PC and non‐neoplastic causes almost matched each other as the main causes of death (48.5% and 47.8%). The results suggest that SPCs appear autonomous from primary PC and reflect incidence and mortality of first cancers in general. SPC was the most common cause of death in patients with SPC; close to half of the patients died due to SPC. For improved survival in PC patients, prevention and early detection of SPCs would be important, and the present results suggest that risk factors for SPC in PC are the same as those for first cancer in general.

## 1 INTRODUCTION

Survival rates of cancer have improved whereby the likelihood for second primary cancers (SPCs) has increased.^1^, ^2^ As prostate cancer (PC) is the most common cancer in males in developed countries, and as survival rates associated with PC are good, SPCs are diagnosed in many patients.^3^ Many studies have reported on SPCs in PC patients with some consistency in increased risk of bladder, kidney, and thyroid cancers.^4^, ^5^, ^6^, ^7^ In Sweden and Germany, SPCs following PC account for 22.5% and 16.9% of all SPCs, respectively.^8^ Paradoxically, in Swedish men, SPCs after PC would collectively rank as the third most frequent cancer type after PC and colorectal cancer but before lung cancer.^9^ A Swedish study, which analyzed deaths in patients with PC until the year 2006, found that SPCs ranked third after PC and myocardial infarctions among the underlying causes of death.^10^ In cancers that have good survival rates, such as Hodgkin lymphoma and testicular cancer, SPCs have become the main cause of death.^1^, ^11^, ^12^ It can be assumed that higher‐order multiple primaries (3rd, 4th, etc. primary cancers) increase along with SPCs although occurrence of multiple primaries is often associated with genetic predisposition in the context of cancer syndromes.^1^, ^11^, ^13^, ^14^ The suspected iatrogenic cause of SPC in PC patients has been radiotherapy but the documented effects have mainly been limited to long follow‐up times in organs of anatomic proximity, mainly the bladder and the rectum.^2^, ^15^, ^16^

In this study, we investigated the risks for and mortality in SPCs in patients with PC. The resource used was the Swedish Family‐Cancer Database that includes all cancers from the national cancer registry and deaths from the cause of death register since 1958. The effect of SPCs as the cause of deaths in PC patients was remarkably high and any attempt to increase survival in patients with PC needs to face the challenge of multiple primaries.

## 2 METHODS

Data for the present study were obtained from the Swedish Family‐Cancer Database which includes information concerning the residents of Sweden organized in families and covers more than a century.^17^ Individuals were linked to the national cancer registry for the first and any subsequent cancers.^17^ The database records cancers according to the International Classification of Diseases 7th revision (ICD‐7) and later revisions. To the end of 2015, more than two million cancers were recorded among 16.1 million individuals. We identified newly diagnosed PC patients from January 1, 2001, until December 31, 2010, and followed these patients from 2001 until the end of 2015 for diagnosis of any of the 28 different SPCs; only invasive cancers were considered. For some analyses, prostate‐specific antigen (PSA)—detected PC was distinguished using the tumor, node, metastasis (TNM) classification code T1c. Note, Sweden has no organized national PC screening programs and T1c data thus refer to findings after opportunistic screening or clinical examinations. Some data on TNM for PC were available from 2001 onwards but case numbers in the first year were low. The follow‐up was terminated at diagnosis of a multiple primary, emigration, death, or December 31, 2015, whichever occurred earliest. Causes of death were available in the database as obtained from the national causes of death register.^18^ Numbers of primary cancers were obtained from Swedish cancer statistics for 70‐ to 79‐year‐old men for the years 2009 and 2012 and were calculated as annual means^9^, ^19^; the selected age bracket matched the diagnostic age of SPCs after PC.

Relative risks (RRs) for SPC were estimated comparing risk (incident rate) of SPCs among PC survivors against risk of that cancer as the first primary in the general population. Waiting time distribution with Poisson assumption was employed to estimate RRs and corresponding confidence intervals (CIs) for 5%, 1%, and 0.1% levels of significance. Generalized linear multivariate model was used with regressors including age group, calendar period, residential area, and socioeconomic status adjusted for potential confounding.

Analyses of causes of death were considered as the underlying cause of death reported in accordance with the ICD‐10 codes. All cancer‐related deaths were stratified into PC, SPC, and “other neoplasia,” which includes cancers diagnosed at the issue of death certificates, referred to “death certificate notifications”.^18^, ^20^, ^21^ These notifications are not used by the Swedish Cancer Registry to complement cancer data, opposite to the other Nordic Cancer Registries.^18^, ^20^, ^21^ It has been found that the notifications often included multiple cancers and cancer of unknown primary (CUP). In our previous studies, we have used these as information on metastases.^22^, ^23^ If the death certificate notification matched the organ site of the reported primary cancer, it was classified to that site but in most cases such an assignment could not be made and the classification was to “other neoplasia.” In some analyses, higher‐order primary cancers were classified separately. Other non‐neoplastic causes of death were reported as “other causes.” All statistical analyses were done with R version 3.4 and SAS version 9.4.

This project was done under approved by the IRB of the Lund University, with a waiver for informed consent.

## 3 RESULTS

Among a total of 4.26 million men, 76,614 were diagnosed with PC at the median age of 71 years between 2001 and 2010 (Table 1). Among them, 8659 (11.3%) received a subsequent diagnosis of SPC when followed to 2015; the median follow‐up time was 4 years. Third, primary cancers were diagnosed in 908 patients, fourth in 138 patients, and fifth in 44 patients, which were 10.5, 1.6, and 0.5% of SPCs, respectively.

**Table 1:** Demographical characteristics of males diagnosed with prostate cancer between 2001 and 2010 in Sweden

Total no. of males followed	4 256 259	
Summary of cases		
I. PC diagnosis (2001‐2010)	76 614	
II. Median age of PC diagnosis	71 y	
III. SPC diagnosis after PC (2001‐2015)	8659 (11.3%)	
IV. Median follow‐up time until SPC diagnosis after PC	4 y	
3rd and higher‐order primaries (2001‐2015)		
V. 3rd primary cancer diagnosis	908 (10.5%)	
VI. 4th primary cancer diagnosis	138 (1.6%)	
VII. 5th primary cancer diagnosis	44 (0.5%)	
Summary of deaths		
VIII. Total no. of deaths among PC patients (2001‐2015)	33 259 (43.4%)	
IX. Total deaths among SPC patients after PC (2001‐2015)	4929 (56.9%)	
Summary of causes of death	All PC patients	PC patients with SPC
a. Death due to PC	14 623 (44.0%)	891 (18.1%)
b. Death due to SPC	2356 (7.1%)	2356 (47.8%)
c. Death due to other neoplastic diseases	1652 (4.9%)	589 (11.9%)
d. Death due to other cause	14 628 (44.0%)	1093 (27.2%)

PC, prostate cancer; SPC, second primary cancer; y, years.

Relative risks for SPCs compared to the risk of respective first cancers are shown in Table 2, which includes SPCs with at least 20 diagnoses but even the rare SPCs were included in the total (last line). The overall RR for SPCs in PSA–detected PC (27.2% of all PC) was marginally lower (1.28) than that in other PC (1.34). RRs for only three sites showed a significant difference (CIs were close to non‐overlapping) between PSA detected and other PC; lung cancer risk was lower and melanoma risk was higher in PSA—detected PC; and bladder cancer risk was higher in other PC. The median diagnostic age for PSA—detected PC was 67 years compared to 73 years for other PC.

**Table 2:** Risk of second primary cancer in prostate cancer survivors according to PSA testing of by other means

Cancer	PSA testing (T1c)			Other diagnosis (other T)			All		
	N	RR	95% CI	N	RR	95% CI	N	RR	95% CI
UAT	78	**1.28**	1.02‐1.60	155	1.09	0.93‐1.28	233	**1.15**	1.01‐1.31
Esophagus	33	0.86	0.61‐1.21	87	0.93	0.75‐1.15	120	0.90	0.75‐1.09
Stomach	53	0.87	0.66‐1.14	171	1.03	0.88‐1.20	224	0.99	0.86‐1.13
Small intestine	22	**1.68**	1.10‐2.57	44	**1.44**	1.06‐1.96	66	***1.52***	1.18‐1.95
Colorectum	458	***1.30***	1.19‐1.43	1178	***1.32***	1.25‐1.40	1636	***1.32***	1.25‐1.39
Liver	41	**0.72**	0.53‐0.98	117	***0.83***	0.69‐1.00	158	***0.80***	0.68‐0.94
Pancreas	78	1.17	0.94‐1.47	170	1.12	0.96‐1.31	248	**1.14**	1.00‐1.30
Lung	187	***0.79***	0.68‐0.91	599	1.04	0.96‐1.13	786	0.97	0.90‐1.04
Other male genitals	13	1.30	0.75‐2.26	29	1.16	0.80‐1.69	42	1.20	0.88‐1.64
Kidney	115	***1.72***	1.43‐2.07	220	***1.47***	1.28‐1.68	335	***1.55***	1.39‐1.73
Bladder	273	***1.38***	1.23‐1.56	901	***1.71***	1.60‐1.83	1174	***1.62***	1.53‐1.72
Melanoma	201	***1.75***	1.52‐2.01	382	***1.44***	1.30‐1.60	583	***1.54***	1.41‐1.67
Skin (SCC)	271	***1.45***	1.28‐1.63	956	***1.63***	1.52‐1.74	1227	***1.58***	1.49‐1.68
Eye	8	1.25	0.62‐2.52	14	0.99	0.58‐1.69	22	1.08	0.70‐1.65
Nervous system	62	**1.31**	1.02‐1.69	121	***1.27***	1.06‐1.53	183	***1.28***	1.11‐1.49
Thyroid gland	12	**1.86**	1.05‐3.30	31	***2.10***	1.46‐3.03	43	***2.03***	1.48‐2.78
Endocrine glands	23	1.19	0.79‐1.80	62	***1.55***	1.20‐2.01	85	***1.44***	1.15‐1.79
Connective tissue	14	0.99	0.58‐1.68	44	1.23	0.91‐1.67	58	1.16	0.89‐1.52
NHL	123	***1.27***	1.06‐1.52	297	***1.25***	1.11‐1.41	420	***1.26***	1.14‐1.39
Multiple myeloma	67	***1.54***	1.21‐1.96	128	1.19	1.00‐1.43	195	***1.29***	1.12‐1.50
Leukemia	119	***1.42***	1.19‐1.71	268	***1.26***	1.12‐1.43	387	***1.31***	1.18‐1.45
CUP	67	0.99	0.78‐1.26	224	***1.26***	1.10‐1.44	291	***1.19***	1.05‐1.34
Total	2350	***1.28***	1.23‐1.33	6299	***1.34***	1.30‐1.37	8649	***1.32***	1.29‐1.35

CI, confidence interval; CUP, cancer of unknown primary; N, total frequency; NHL, non‐Hodgkin lymphoma; PSA, prostate‐specific antigen; RR, relative risk; SCC, squamous cell carcinoma; UAT, upper aero‐digestive tract;

Bold, italics, underline indicate 5%, 1% and 0.1% level of significance, respectively.

**Figure 1:**
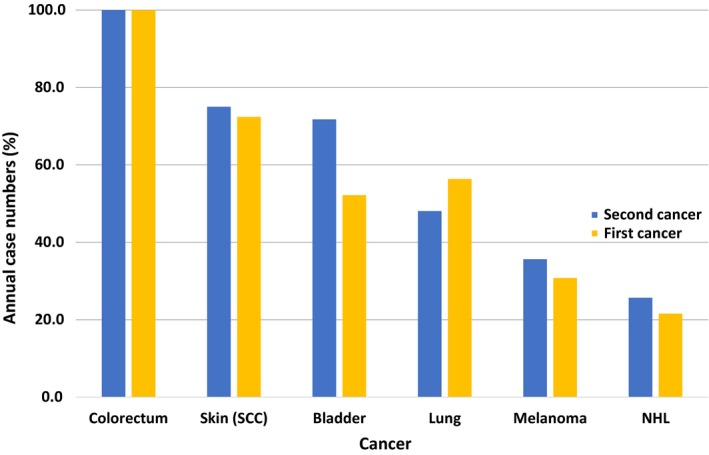
Comparison of proportions of annual case numbers for the six most incident second primary cancers in prostate cancer survivors and that of the first cancers in the Swedish cancer registry for 70‐ to 79‐year‐old men diagnoses in years 2009 and 2012. The annual cases numbers in the cancer registry ranged from 1192 in colorectal cancer to 257 in non‐Hodgkin lymphoma. SCC, squamous cell carcinoma; NHL, non‐Hodgkin lymphoma

For all SPCs, the overall RR was 1.32, and RRs for 15 individual SPCs were increased (Table 2). The highest RRs were observed for thyroid (2.03) and bladder (1.62) cancers; for skin (squamous cell carcinoma), kidney and small intestinal cancers and for melanoma, the RRs were between 1.52 and 1.58. Ranking by numbers of patients with SPC was topped by colorectal, skin, and bladder cancers—all exceeded 1000 cases—and followed by lung cancer, melanoma, and non‐Hodgkin lymphoma. The ranking of SPCs was compared to the incidence ranking for first primary cancer in the Swedish cancer registry for 70‐ to 79‐year‐old men (Figure 1); the only exception the reversed order of bladder and lung cancers was reversed as presented in: colorectum (1192 patients, mean number of diagnoses in years 2009 and 2012), skin (863), lung (672), bladder (622), melanoma (367), and non‐Hodgkin lymphoma (257); the number of patients with PC was 3038.

We analyzed SPC risks by follow‐up time which showed increasing overall RRs from 1.27 (<1 year of follow‐up) to 1.41 (>10 years; Table S1). RRs were initially high for bladder and kidney cancers while the opposite trend was found for skin cancer, nervous system tumors, and leukemia. We further analyzed risks of SPC by diagnostic age for PC (Table S2). A U‐shaped trend was observed for higher RRs in both the young and very elderly patients; the overall RR for SPC was 5.70 when PC was diagnosed at age <60 years and 3.71 when PC was diagnosed ≥90. SPCs with the high risks in young patients were thyroid (11.65) and bladder (11.42) cancers.

Of 76 614 PC patients, 33 259 (43.4%) had died, while among patients with SPC 4929 (56.9%) had died (Table 1 lower part). The cause of death for all PC was PC in 44.0% of all deaths, which was equally high as for other (non‐neoplastic) causes. In patients with SPC, SPC was the dominant cause of death (47.8%), followed by other causes (27.2%) and PC (18.1%).

**Figure 2:**
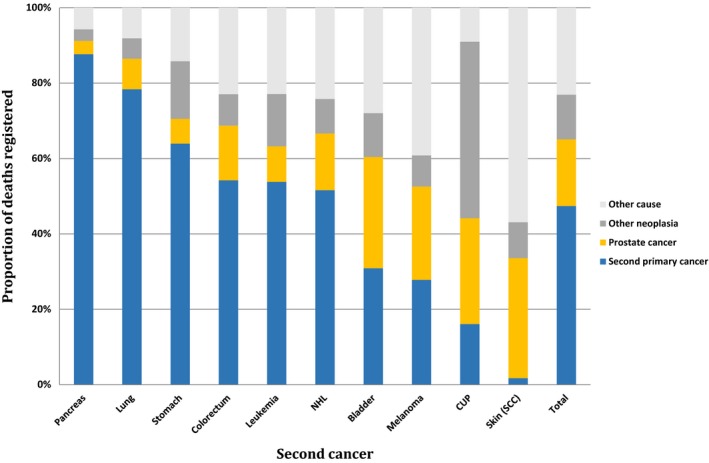
Distribution of causes of death among different second primary cancer sites for PC survivors. SCC, squamous cell carcinoma; CUP, cancer of unknown primary; NHL, non‐Hodgkin lymphoma

Causes of death in PC with SPC depended on the type of SPC (full data are shown in Table S3). The results are summarized for the 10 most common SPCs and for all cancers (total) in Figure 2. Pancreatic, lung, and stomach cancers were the most fatal SPCs while CUP and squamous cell skin cancer were the least fatal SPCs. However, for CUP other than neoplasia accounted for 46.8% of deaths and very likely these included a large proportion of CUP cases (see methods).

Age‐group‐specific comparison of causes of death for PC patients, with and without SPC, is described in Table 3. For patients with SPC, the second primaries were the main cause of death in age groups below 80 years, and the proportion decreased from 68.3% at age group <60 years to 20.4% at age group >90 years. Other (non‐neoplastic) causes were the main cause of death in the oldest age groups. For PC patients without SPC, PC was the main cause of death below age 80 years but other causes overtook it in higher age groups. Notably, other types of neoplasia were three to four times more common causes of death in PC patients with SPC (11.9%) compared to PC patients without SPC (3.7%).

**Table 3:** Age‐group‐specific comparison of causes of death for prostate cancer patients, with and without second primary cancer

	Age group	Causes of death				Total
		Prostate cancer	SPC	Other neoplasia	Other cause	
		N (%)	N (%)	N (%)	N (%)	N
PC patients with SPC	< 60	26 (12.7)	140 (68.3)	25 (12.2)	14 (6.8)	205
	60‐69	191 (13.9)	829 (60.5)	180 (13.1)	171 (12.5)	1371
	70‐79	411 (19.1)	1007 (46.6)	262 (12.1)	480 (22.2)	2160
	80‐89	250 (21.9)	369 (32.5)	115 (10.1)	405 (35.6)	1139
	**≥**90	13 (24.1)	11 (20.4)	7 (12.9)	23 (42.6)	54
	Total	891 (18.1)	2356 (47.8)	589 (11.9)	1093 (22.2)	4929
PC patients without SPC	<60	759 (71.3)	‐	34 (3.2)	271 (25.5)	1064
	60‐69	2861 (58.3)	‐	177 (3.6)	1868 (38.1)	4906
	70‐79	5481 (48.8)	‐	439 (3.9)	5307 (47.3)	11 227
	80‐89	4223 (41.7)	‐	379 (3.7)	5535 (54.6)	10 137
	**≥** 90	408 (41.0)	‐	34 (3.4)	554 (55.6)	996
	Total	13 732 (48.5)	‐	1063 (3.7)	13 535 (47.8)	28 330

N (%), frequency and percentage; SPC, second primary cancer; SPC^+^/SPC^‐^: Individuals diagnosed with SPC or not.

Age‐group‐specific analysis was carried out for individual cancers, and data are shown for all cancers in Table S4. The general observations include that fatal SPCs were the main cause of deaths at all ages, while for benign SPCs PC and other causes, particularly in older individuals, were the main cause of mortality. Data on three SPCs, which contributed the most common causes (43.8%) of all deaths, are shown in Table 4. When lung and colorectal cancers were SPCs, these cancers were the most prominent causes of death in all age groups. Second, primary bladder cancer was the most common cause of death until the age of 80 years while at higher ages it was overtaken by other cancers.

**Table 4:** Causes of death in prostate cancer patients with second primary cancer in the colorectum, lung, and bladder at age groups of prostate cancer diagnosis

Cancer	Age group	Causes of death				Total
		Prostate cancer	SPC	Other neoplasia	Other cause	
		N (%)	N (%)	N (%)	N (%)	N
Colorectum	<60	7 (23.3)	21 (70.0)	‐	2 (6.7)	30
	60‐69	22 (11.1)	135 (68.2)	10 (5.1)	31 (15.7)	198
	70‐79	62 (14.3)	237 (54.6)	34 (7.8)	101 (23.3)	434
	80‐89	40 (16.6)	99 (41.1)	29 (12.0)	73 (30.3)	241
	≥90	2 (16.7)	4 (33.3)	3 (25.0)	3 (25.0)	12
	Total	133 (14.50)	496 (54.2)	76 (8.3)	210 (23.0)	915
Lungs	<60	1 (2.4)	37 (90.2)	3 (7.3)	‐	41
	60‐69	10 (4.5)	188 (85.5)	9 (4.1)	13 (5.9)	220
	70‐79	30 (9.8)	230 (75.4)	19 (6.2)	26 (8.5)	305
	80‐89	13 (13.3)	66 (67.3)	5 (5.1)	14 (14.3)	98
	≥90	‐	1 (50.0)	‐	1 (50.0)	2
	Total	54 (8.1)	522 (78.4)	36 (5.4)	54 (8.1)	666
Bladder	<60	1 (5.9)	14 (82.4)	‐	2 (11.8)	17
	60‐69	33 (28.9)	46 (40.4)	13 (11.4)	22 (19.3)	114
	70‐79	77 (30.8)	79 (31.6)	28 (11.2)	66 (26.4)	250
	80‐89	57 (32.8)	38 (21.8)	21 (12.1)	58 (33.3)	174
	≥90	2 (9.5)	1 (4.8)	5 (23.8)	13 (61.9)	21
	Total	170 (29.5)	178 (30.9)	67 (11.6)	161 (28.0)	576

N (%), frequency, and percentage; SPC, second primary cancer.

## 4 DISCUSSION

The present study focused on PC diagnosed in the 10‐year period from 2001 to 2010, and the follow‐up was extended to the end of 2015. The reason for selecting these periods was that around that time the rapidly increasing incidence of PC practically stalled and the 5‐year extension to follow‐up allowed the possibility to catch subsequent events occurring up to 15 years after PC diagnosis.^21^ As the median follow‐up time from PC diagnosis to SPC was 4 years, whereby we can assume that we were able to identify most SPCs among this population. The present results revealed novel features about demography of SPC after PC. SPC was diagnosed in 11.3% of PC patients, and the proportion was not different (10.5%) for third primary cancers found in SPC patients. Incidence ranking for SPCs followed the incidence of each specific cancer as a first cancer, with the exception that ranks three and four shifted places: Colorectal and skin cancer were the two most common cancers followed by bladder cancer, which was more common than lung cancer as SPC but the order was reversed among first cancers. The paradoxical suggestion from similar incidence rates may be that the etiology is also shared. Tobacco smoking is not a risk factor for PC but it may be an equally important cause for second lung cancer as it is for first lung cancer.^24^

PSA—detected PC accounted for 27.2% of all PC in this population, and the overall RR for SPC was only marginally lower (1.28) in PSA detected compared to other PC (1.34). The implication may be that the risk of SPC is independent of the stage of PC. RRs in the two types of PC differed only for three SPCs. Melanoma risk was higher in PSA–detected PC while lung and bladder cancer risks were higher in other PC. These differences have likely explanations. Blood in urine will lead to a urological examination, and this scenario is likely for other PC. Low risk of lung cancer and high risk of melanoma point to socioeconomic correlates; for these particular cancers, educational and socioeconomic gradients were the steepest in Sweden.^25^, ^26^ Socioeconomic factors are likely to reflect health consciousness and active participation in health check‐ups.

Mortality patterns of PC patients were distinct depending on the presence or absence of SPC. Among patients with SPC, 47.8% died as a result of that cancer, followed by other causes (22.2%) and PC (18.1%). For patients without SPC, PC and other causes almost matched each other as the main causes of death (48.5% and 47.8%). Other types of neoplasia, which constituted “death certificate notifications,” accounted for 11.9% of deaths in PC with SPC compared to 3.8% in patients without SPC. The difference was relatively constant through age groups and thus the known lower completeness of cancer registration at high age could not be the reason.^18^, ^20^, ^21^ We suspect that these other types of neoplasia were metastases originating from first or second primary cancer. Thus, “other neoplasia” will likely contribute to deaths in first and second primaries if the origin of metastasis was known.

The results showed that different SPCs had vastly different consequences to patients’ life expectancy. The majority of patients died due to SPC if this was pancreatic, lung, or stomach cancer while only a few patients died as a result of melanoma and hardly any patients died due to squamous cell skin cancer. This is largely what can be expected based on prognosis in first primary cancer. CUP is a metastatic highly fatal cancer and yet it appeared not fatal as SPC^27^, ^28^, ^29^; there are two likely explanations. In Figure 2, CUP shows by far the largest share (45%) for “other neoplasia” as the cause of death, which probably includes a large number of CUP patients. The other reason is the death registration practice for CUP in Sweden. The cause of death is often recorded as cancer that has caused the patient's death, that is, if the CUP patient was diagnosed with liver metastases, the cause of death could be assigned to liver cancer and not to CUP.^30^

In conclusion, we showed that the risk of SPC was equally high both in PSA detected and other PC, which suggests that SPCs were independent of the stage of PC. The ranking of SPCs was exactly like first cancers among elderly Swedish men, with the exception that bladder and lung cancers shifted orders. Even mortality was governed by the type of SPC, mimicking mortality in first primary cancers. The independent features of SPCs suggest that these were autonomous from PC. SPC was the cause of death in close to half of the patients with SPC. For improved survival in PC, prevention and early detection of SPCs would be important. The risk factors for SPC in PC may be the same as those for first cancer in general.

## CONFLICTS OF INTEREST

The authors have nothing to declare.
