# Supplementary material for: Prostate cancer survivors: Risk and mortality in second primary cancers
Source: Cancer Med. 2018 Oct 1;7(11):5752–9. doi: 10.1002/cam4.1764 (PMC6246949; doi:10.1002/cam4.1764)
Supplement: Supplementary file 1 [file CAM4-7-5752-s001.docx]

|  | **Follow-up time** | | | | | | | | | | | | | | | |
| --- | --- | --- | --- | --- | --- | --- | --- | --- | --- | --- | --- | --- | --- | --- | --- | --- |
|  | **< 1 year** | | | | **1 – 5 years** | | | | **6 – 10 years** | | | | **> 10 years** | | | |
| **Cancer** | **N** | **RR** | **LCI** | **UCI** | **N** | **RR** | **LCI** | **UCI** | **N** | **RR** | **LCI** | **UCI** | **N** | **RR** | **LCI** | **UCI** |
| UAT | 55 | 1.11 | 0.85 | 1.45 | 95 | 1.11 | 0.90 | 1.36 | 73 | **1.27** | 1.01 | 1.61 | 10 | 0.98 | 0.53 | 1.82 |
| Esophagus | 20 | 0.65 | 0.42 | 1.00 | 58 | 1.04 | 0.80 | 1.35 | 34 | 0.88 | 0.63 | 1.23 | 8 | 1.12 | 0.56 | 2.25 |
| Stomach | 46 | 0.90 | 0.68 | 1.21 | 91 | 0.97 | 0.79 | 1.19 | 77 | 1.12 | 0.89 | 1.40 | 10 | 0.74 | 0.40 | 1.37 |
| Small intestine | 9 | 0.90 | 0.47 | 1.74 | 33 | ***1.80*** | 1.27 | 2.56 | 21 | **1.62** | 1.05 | 2.51 | 3 | 1.24 | 0.40 | 3.87 |
| Colorectum | 373 | ***1.36*** | 1.23 | 1.50 | 701 | ***1.36*** | 1.26 | 1.47 | 492 | ***1.30*** | 1.19 | 1.42 | 70 | 0.95 | 0.75 | 1.20 |
| Anus | 4 | 1.27 | 0.47 | 3.41 | 8 | 1.44 | 0.71 | 2.91 | 3 | 0.80 | 0.25 | 2.49 | - | - | - | - |
| Liver | 32 | **0.68** | 0.48 | 0.97 | 61 | **0.74** | 0.57 | 0.95 | 57 | 0.99 | 0.76 | 1.29 | 8 | 0.76 | 0.38 | 1.52 |
| Pancreas | 59 | 1.19 | 0.92 | 1.54 | 97 | 1.06 | 0.87 | 1.30 | 76 | 1.17 | 0.93 | 1.47 | 16 | 1.33 | 0.81 | 2.18 |
| Nose | 2 | 0.67 | 0.17 | 2.68 | 6 | 1.14 | 0.51 | 2.56 | 6 | 1.67 | 0.74 | 3.76 | 1 | 1.54 | 0.22 | 11.02 |
| Lung | 194 | 1.03 | 0.90 | 1.19 | 337 | 0.99 | 0.89 | 1.10 | 217 | 0.90 | 0.79 | 1.03 | 38 | 0.85 | 0.62 | 1.17 |
| Breast | 4 | 1.29 | 0.48 | 3.45 | 5 | 0.90 | 0.37 | 2.18 | 6 | 1.58 | 0.70 | 3.56 | - | - | - | - |
| Testis | 3 | 1.58 | 0.51 | 4.94 | 3 | 1.06 | 0.34 | 3.32 | 3 | 1.97 | 0.63 | 6.20 | - | - | - | - |
| Other male genitals | 10 | 1.25 | 0.67 | 2.33 | 18 | 1.22 | 0.77 | 1.95 | 13 | 1.27 | 0.73 | 2.20 | 1 | 0.51 | 0.07 | 3.66 |
| Kidney | 90 | ***1.78*** | 1.45 | 2.20 | 133 | ***1.45*** | 1.22 | 1.72 | 98 | ***1.54*** | 1.26 | 1.89 | 14 | 1.22 | 0.72 | 2.07 |
| Bladder | 340 | ***2.17*** | 1.95 | 2.41 | 458 | ***1.53*** | 1.40 | 1.68 | 307 | ***1.36*** | 1.21 | 1.52 | 69 | ***1.51*** | 1.19 | 1.92 |
| Melanoma | 97 | 1.13 | 0.93 | 1.39 | 239 | ***1.51*** | 1.33 | 1.72 | 208 | ***1.83*** | 1.59 | 2.10 | 38 | ***1.75*** | 1.27 | 2.41 |
| Skin (SCC) | 175 | 1.15 | 0.99 | 1.33 | 500 | ***1.62*** | 1.48 | 1.77 | 441 | ***1.72*** | 1.56 | 1.89 | 111 | ***1.91*** | 1.58 | 2.30 |
| Eye | 6 | 1.24 | 0.55 | 2.77 | 8 | 0.92 | 0.46 | 1.86 | 7 | 1.19 | 0.56 | 2.51 | 1 | 0.95 | 0.13 | 6.78 |
| Nervous system | 42 | 1.21 | 0.89 | 1.64 | 52 | 0.85 | 0.65 | 1.12 | 73 | ***1.82*** | 1.45 | 2.30 | 16 | ***2.39*** | 1.46 | 3.91 |
| Thyroid gland | 3 | 0.58 | 0.19 | 1.81 | 23 | ***2.56*** | 1.68 | 3.89 | 15 | ***2.49*** | 1.48 | 4.18 | 2 | 1.87 | 0.46 | 7.51 |
| Endocrine glands | 19 | 1.31 | 0.83 | 2.06 | 28 | 1.11 | 0.76 | 1.62 | 35 | ***2.11*** | 1.50 | 2.95 | 3 | 1.06 | 0.34 | 3.31 |
| Bone | 5 | ***3.25*** | 1.33 | 7.93 | 1 | 0.38 | 0.05 | 2.75 | 2 | 1.16 | 0.29 | 4.72 | 1 | 3.28 | 0.45 | 23.60 |
| Connective tissue | 19 | 1.72 | 1.09 | 2.70 | 19 | 0.92 | 0.58 | 1.45 | 16 | 1.05 | 0.64 | 1.73 | 4 | 1.35 | 0.50 | 3.60 |
| NHL | 98 | ***1.32*** | 1.08 | 1.61 | 180 | ***1.30*** | 1.12 | 1.50 | 110 | 1.09 | 0.90 | 1.31 | 31 | **1.58** | 1.11 | 2.25 |
| Hodgkin lymphoma | 2 | 0.63 | 0.16 | 2.53 | 6 | 1.11 | 0.49 | 2.49 | 5 | 1.40 | 0.58 | 3.41 | 1 | 1.67 | 0.23 | 11.90 |
| Multiple myeloma | 54 | ***1.61*** | 1.23 | 2.11 | 81 | **1.29** | 1.04 | 1.61 | 50 | 1.09 | 0.82 | 1.45 | 10 | 1.12 | 0.60 | 2.09 |
| Leukemia | 72 | 1.10 | 0.87 | 1.39 | 154 | ***1.26*** | 1.07 | 1.48 | 126 | ***1.40*** | 1.17 | 1.67 | 35 | ***1.98*** | 1.42 | 2.76 |
| CUP | 66 | 1.17 | 0.92 | 1.49 | 124 | **1.21** | 1.02 | 1.45 | 87 | 1.19 | 0.96 | 1.47 | 14 | 1.00 | 0.59 | 1.69 |
| Total | 1923 | ***1.27*** | 1.22 | 1.33 | 3537 | ***1.29*** | 1.25 | 1.34 | 2670 | ***1.38*** | 1.33 | 1.43 | 517 | ***1.41*** | 1.29 | 1.53 |

**Supplementary Table 1.** Risk of second cancers among prostate cancer survivors diagnosed between 2001 and 2010 stratified over follow up time

Abbreviations:

N, total frequency; RR, relative risk; LCI, lower 95% confidence interval limit; UCI, upper 95% confidence interval limit; UAT, upper aero-digestive tract; SCC, squamous cell carcinoma; NHL, non-Hodgkin lymphoma; CUP, cancer of unknown primary

Bold, italics, underline indicate 5%, 1% and 0.1% level of significance respectively

|  | **< 60** | | | | **60 – 69** | | | | **70 – 79** | | | | **80 – 89** | | | | **≥ 90** | | | |
| --- | --- | --- | --- | --- | --- | --- | --- | --- | --- | --- | --- | --- | --- | --- | --- | --- | --- | --- | --- | --- |
| **Cancer** | **N** | **RR** | **LCI** | **UCI** | **N** | **RR** | **LCI** | **UCI** | **N** | **RR** | **LCI** | **UCI** | **N** | **RR** | **LCI** | **UCI** | **N** | **RR** | **L_CI** | **U_CI** |
| UAT | 27 | ***4.12*** | 2.81 | 6.03 | 102 | ***2.34*** | 1.91 | 2.86 | 67 | ***1.50*** | 1.17 | 1.92 | 36 | ***1.73*** | 1.24 | 2.41 | 1 | 2.60 | 0.36 | 18.62 |
| Esophagus | 11 | ***4.79*** | 2.63 | 8.72 | 42 | ***1.83*** | 1.34 | 2.49 | 47 | **1.38** | 1.03 | 1.85 | 18 | 1.22 | 0.76 | 1.95 | 2 | **9.86** | 2.40 | 40.44 |
| Stomach | 6 | 2.09 | 0.93 | 4.67 | 78 | ***2.65*** | 2.11 | 3.34 | 93 | ***1.66*** | 1.35 | 2.05 | 45 | **1.35** | 1.00 | 1.82 | 2 | 2.93 | 0.73 | 11.81 |
| Small intestine | 5 | ***5.39*** | 2.21 | 13.13 | 29 | ***3.16*** | 2.16 | 4.63 | 21 | ***1.99*** | 1.27 | 3.11 | 11 | ***2.38*** | 1.29 | 4.38 | - | - | - | - |
| Colorectum | 98 | ***6.50*** | 5.31 | 7.95 | 540 | ***3.03*** | 2.78 | 3.31 | 711 | ***2.10*** | 1.94 | 2.27 | 278 | ***1.85*** | 1.64 | 2.09 | 9 | ***3.79*** | 1.96 | 7.32 |
| Anus | 1 | 2.36 | 0.33 | 17.01 | 7 | **2.31** | 1.07 | 4.96 | 3 | 1.01 | 0.32 | 3.23 | 3 | 2.65 | 0.82 | 8.53 | 1 | **84.38** | 9.31 | 765.07 |
| Liver | 11 | ***3.22*** | 1.77 | 5.84 | 58 | ***1.91*** | 1.47 | 2.49 | 69 | 1.23 | 0.97 | 1.57 | 20 | 0.91 | 0.58 | 1.41 | - | - | - | - |
| Pancreas | 18 | ***4.84*** | 3.03 | 7.73 | 118 | ***3.15*** | 2.61 | 3.80 | 90 | ***1.46*** | 1.18 | 1.80 | 21 | 1.18 | 0.76 | 1.82 | 1 | 6.28 | 0.87 | 45.58 |
| Nose | 2 | ***6.93*** | 1.69 | 28.49 | 4 | 1.90 | 0.70 | 5.20 | 4 | 1.35 | 0.49 | 3.70 | 5 | **3.21** | 1.29 | 8.03 | - | - | - | - |
| Lung | 53 | ***5.71*** | 4.34 | 7.51 | 295 | ***2.27*** | 2.02 | 2.55 | 334 | ***1.44*** | 1.29 | 1.61 | 102 | **1.27** | 1.05 | 1.55 | 2 | 2.64 | 0.65 | 10.61 |
| Breast | 2 | ***8.17*** | 1.98 | 33.83 | 7 | ***3.94*** | 1.81 | 8.59 | 4 | 1.29 | 0.47 | 3.53 | 2 | 1.21 | 0.30 | 4.95 | - | - | - | - |
| Testis | 4 | ***3.85*** | 1.43 | 10.33 | 2 | 1.11 | 0.27 | 4.53 | 1 | 0.73 | 0.10 | 5.32 | 2 | **6.98** | 1.51 | 32.37 | - | - | - | - |
| Other male genitals | 2 | 2.94 | 0.73 | 11.89 | 10 | 1.54 | 0.82 | 2.89 | 21 | ***3.02*** | 1.92 | 4.75 | 8 | 1.79 | 0.88 | 3.64 | 1 | 7.06 | 0.97 | 51.45 |
| Kidney | 33 | ***6.96*** | 4.91 | 9.86 | 157 | ***3.65*** | 3.09 | 4.30 | 121 | ***2.04*** | 1.69 | 2.45 | 24 | 1.41 | 0.94 | 2.12 | - | - | - | - |
| Bladder | 79 | ***11.42*** | 9.09 | 14.35 | 398 | ***4.52*** | 4.07 | 5.02 | 469 | ***2.55*** | 2.32 | 2.81 | 210 | ***2.03*** | 1.76 | 2.33 | 18 | ***9.44*** | 5.90 | 15.10 |
| Melanoma | 57 | ***5.33*** | 4.10 | 6.94 | 243 | ***3.35*** | 2.93 | 3.82 | 208 | ***2.21*** | 1.91 | 2.54 | 74 | ***1.97*** | 1.56 | 2.50 | 1 | 1.19 | 0.17 | 8.51 |
| Skin (SCC) | 40 | ***9.62*** | 6.99 | 13.24 | 320 | ***5.59*** | 4.96 | 6.30 | 545 | ***3.35*** | 3.06 | 3.67 | 309 | ***2.16*** | 1.93 | 2.43 | 13 | ***2.55*** | 1.48 | 4.41 |
| Eye | 1 | 1.68 | 0.23 | 12.01 | 12 | ***2.72*** | 1.51 | 4.89 | 6 | 1.14 | 0.50 | 2.59 | 3 | 1.89 | 0.59 | 6.01 | - | - | - | - |
| Nervous system | 34 | ***5.57*** | 3.96 | 7.84 | 86 | ***2.37*** | 1.90 | 2.94 | 56 | ***1.61*** | 1.23 | 2.12 | 7 | 0.90 | 0.43 | 1.90 | - | - | - | - |
| Thyroid gland | 9 | ***11.65*** | 5.94 | 22.87 | 17 | ***3.89*** | 2.35 | 6.42 | 13 | ***2.38*** | 1.34 | 4.23 | 4 | 1.95 | 0.71 | 5.34 | - | - | - | - |
| Endocrine glands | 12 | ***5.03*** | 2.83 | 8.92 | 38 | ***2.74*** | 1.97 | 3.81 | 29 | ***1.81*** | 1.24 | 2.64 | 6 | 1.84 | 0.81 | 4.18 | - | - | - | - |
| Bone | 0 | ***5.03*** | 2.83 | 8.92 | 5 | ***3.54*** | 1.42 | 8.86 | 4 | **3.83** | 1.33 | 11.02 | - | - | - | - | - | - | - | - |
| Connective tissue | 6 | ***6.78*** | 3.00 | 15.31 | 14 | **1.84** | 1.07 | 3.14 | 24 | ***1.98*** | 1.30 | 3.00 | 12 | **1.87** | 1.05 | 3.35 | 2 | **12.80** | 3.10 | 52.86 |
| NHL | 23 | ***3.76*** | 2.49 | 5.69 | 161 | ***3.08*** | 2.62 | 3.62 | 173 | ***2.01*** | 1.72 | 2.35 | 62 | ***1.59*** | 1.24 | 2.05 | 1 | 1.66 | 0.23 | 11.85 |
| Hodgkin lymphoma | 1 | 1.88 | 0.26 | 13.44 | 7 | **2.64** | 1.23 | 5.70 | 4 | 1.16 | 0.43 | 3.18 | 2 | 1.72 | 0.42 | 7.11 | - | - | - | - |
| Multiple myeloma | 10 | ***4.23*** | 2.26 | 7.93 | 75 | ***3.35*** | 2.64 | 4.24 | 85 | ***2.19*** | 1.75 | 2.73 | 25 | 1.41 | 0.95 | 2.11 | - | - | - | - |
| Leukemia | 27 | ***5.66*** | 3.86 | 8.30 | 155 | ***3.83*** | 3.24 | 4.52 | 153 | ***1.97*** | 1.67 | 2.33 | 51 | **1.40** | 1.06 | 1.86 | 1 | 1.83 | 0.26 | 13.08 |
| CUP | 24 | ***6.73*** | 4.48 | 10.13 | 119 | ***3.50*** | 2.90 | 4.23 | 116 | ***1.90*** | 1.57 | 2.30 | 30 | 0.88 | 0.61 | 1.26 | 2 | 3.55 | 0.88 | 14.32 |
| Total | 602 | ***5.70*** | 5.26 | 6.18 | 3127 | ***3.01*** | 2.90 | 3.13 | 3489 | ***1.95*** | 1.88 | 2.02 | 1374 | ***1.66*** | 1.58 | 1.76 | 57 | ***3.71*** | 2.86 | 4.82 |

**Supplementary Table 2.** Risk of SPCs among PC survivors diagnosed between 2001 and 2010 stratified over age at PC diagnosis

Abbreviations:

N, total frequency; RR, relative risk; LCI, lower 95% confidence interval limit; UCI, upper 95% confidence interval limit; UAT, upper aero-digestive tract; SCC, squamous cell carcinoma; NHL, non-Hodgkin lymphoma; CUP, cancer of unknown primary

Bold, italics, underline indicate 5%, 1% and 0.1% level of significance respectively

**Supplementary Table 3.** Causes of death of prostate cancer patients diagnosed between with a second primary cancer

| **Cancer** | **Causes of death** | | | | | | | | |
| --- | --- | --- | --- | --- | --- | --- | --- | --- | --- |
|  | **Total** | **Prostate** | | **Second primary cancer** | | **Other neoplasia** | | **Other cause** | |
|  |  | **N** | **%** | **N** | **%** | **N** | **%** | **N** | **%** |
| UAT | **104** | 21 | 19.09 | 43 | 39.09 | 10 | 9.09 | 30 | 27.27 |
| Esophagus | **97** | 7 | 7.22 | 73 | 75.26 | 8 | 8.25 | 9 | 9.28 |
| Stomach | **197** | 13 | 6.60 | 126 | 63.96 | 30 | 15.23 | 28 | 14.21 |
| Small intestine | **36** | 7 | 17.07 | 14 | 34.15 | 3 | 7.32 | 12 | 29.27 |
| Colorectum | **915** | 133 | 14.54 | 496 | 54.21 | 76 | 8.31 | 210 | 22.95 |
| Anus | **9** | 3 | 33.33 | 1 | 11.11 | 3 | 33.33 | 2 | 22.22 |
| Liver | **120** | 14 | 11.02 | 85 | 66.93 | 11 | 8.66 | 10 | 7.87 |
| Pancreas | **227** | 8 | 3.52 | 199 | 87.67 | 7 | 3.08 | 13 | 5.73 |
| Nose | **7** | 1 | 9.09 | 1 | 9.09 | 4 | 36.36 | 1 | 9.09 |
| Lung | **666** | 54 | 8.11 | 522 | 78.38 | 36 | 5.41 | 54 | 8.11 |
| Breast | **5** | 2 | 40.00 | 1 | 20.00 | 1 | 20.00 | 1 | 20.00 |
| Testis | **5** | 2 | 40.00 | 2 | 40.00 | - | - | 1 | 20.00 |
| Other male genitals | **21** | 13 | 59.09 | 3 | 13.64 | 1 | 4.55 | 4 | 18.18 |
| Kidney | **164** | 30 | 18.29 | 78 | 47.56 | 18 | 10.98 | 38 | 23.17 |
| Bladder | **576** | 170 | 29.51 | 178 | 30.90 | 67 | 11.63 | 161 | 27.95 |
| Melanoma | **194** | 48 | 24.74 | 54 | 27.84 | 16 | 8.25 | 76 | 39.18 |
| Skin (SCC) | **515** | 164 | 31.84 | 9 | 1.75 | 49 | 9.51 | 293 | 56.89 |
| Eye | **11** | 3 | 27.27 | 1 | 9.09 | 2 | 18.18 | 5 | 45.45 |
| Nervous system | **114** | 13 | 10.57 | 74 | 60.16 | 11 | 8.94 | 16 | 13.01 |
| Thyroid gland | **20** | 1 | 4.76 | 13 | 61.90 | 2 | 9.52 | 4 | 19.05 |
| Endocrine glands | **26** | 10 | 33.33 | - | - | 2 | 6.67 | 14 | 46.67 |
| Bone | **7** | 1 | 14.29 | 4 | 57.14 | 1 | 14.29 | 1 | 14.29 |
| Connective tissue | **40** | 10 | 25.00 | 10 | 25.00 | 10 | 25.00 | 10 | 25.00 |
| NHL | **219** | 33 | 15.07 | 113 | 51.60 | 20 | 9.13 | 53 | 24.20 |
| Hodgkin lymphoma | **8** | 1 | 12.50 | 6 | 75.00 | - | - | 1 | 12.50 |
| Multiple myeloma | **136** | 21 | 15.00 | 87 | 62.14 | 3 | 2.14 | 25 | 17.86 |
| Leukemia | **223** | 21 | 9.42 | 120 | 53.81 | 31 | 13.90 | 51 | 22.87 |
| CUP | **267** | 75 | 28.09 | 43 | 16.10 | 125 | 46.82 | 24 | 8.99 |
| Total | **4929** | 879 | 17.69 | 2356 | 47.40 | 588 | 11.83 | 1147 | 23.08 |

Abbreviations:

N, total frequency; %, percentage frequency; UAT, upper aero-digestive tract; SCC, squamous cell carcinoma; NHL, non-Hodgkin lymphoma; CUP, cancer of unknown primary

**Supplementary Table 4.** Cancer specific distribution of causes of death subject to diagnosis of a second primary cancer after prostate cancer stratified over age at first cancer diagnosis

|  | **Cause of death** | | | | | | | | | | |
| --- | --- | --- | --- | --- | --- | --- | --- | --- | --- | --- | --- |
| **Cancer** | **Age group** | **Prostate cancer** | | **Second primary cancer** | | **Other neoplasia** | | **Other cause** | | **Total** |  |
|  |  | **N** | **%** | **N** | **%** | **N** | **%** | **N** | **%** | **N** |  |
| Upper aero-digestive tract | **< 60** | 1 | 16.7 | 1 | 16.7 | 3 | 50.0 | 1 | 16.7 | 6 |  |
|  | **60 – 69** | 6 | 18.8 | 17 | 53.1 | 3 | 9.4 | 6 | 18.8 | 32 |  |
|  | **70 – 79** | 5 | 15.2 | 15 | 45.5 | 3 | 9.1 | 10 | 30.3 | 33 |  |
|  | **80 – 89** | 8 | 25.0 | 10 | 31.3 | 1 | 3.1 | 13 | 40.6 | 32 |  |
|  | **≥ 90** | 1 | 100.0 | - | - | - | - | - | - | 1 |  |
|  | **Total** | 21 | 20.2 | 43 | 41.3 | 10 | 9.6 | 30 | 28.8 | 104 |  |
|  | | | | | | | | | | |  |
| Esophagus | **<60** | - | - | 6 | 85.7 | - | - | 1 | 14.3 | 7 |  |
|  | **60 – 69** | 4 | 12.9 | 25 | 80.6 | - | - | 1 | 3.2 | 31 |  |
|  | **70 – 79** | 3 | 7.5 | 28 | 70.0 | 3 | 7.5 | 6 | 15.0 | 40 |  |
|  | **80 – 89** | - | - | 12 | 70.6 | 4 | 23.5 | 1 | 5.9 | 17 |  |
|  | **≥ 90** | - | - | 2 | 100.0 | - | - | - | - | 2 |  |
|  | **Total** | 7 | 7.2 | 73 | 75.3 | 8 | 8.2 | 9 | 9.3 | 97 |  |
|  | | | | | | | | | | |  |
| Stomach | **<60** | 1 | 20.0 | 4 | 80.0 | - | - | - | - | 5 |  |
|  | **60 – 69** | 2 | 3.3 | 46 | 75.4 | 8 | 13.1 | 5 | 8.2 | 61 |  |
|  | **70 – 79** | 6 | 7.1 | 49 | 57.6 | 14 | 16.5 | 16 | 18.8 | 85 |  |
|  | **80 – 89** | 4 | 9.1 | 25 | 56.8 | 8 | 18.2 | 7 | 15.9 | 44 |  |
|  | **≥ 90** | - | - | 2 | 100.0 | - | - | - | - | 2 |  |
|  | **Total** | 13 | 6.6 | 126 | 64.0 | 30 | 15.2 | 28 | 14.2 | 197 |  |
|  | | | | | | | | | | |  |
| Small intestine | **<60** | - | - | - | - | - | - | - | - | 0 |  |
|  | **60 – 69** | 2 | 13.3 | 11 | 73.3 | 1 | 6.7 | 1 | 6.7 | 15 |  |
|  | **70 – 79** | 2 | 16.7 | 1 | 8.3 | 1 | 8.3 | 8 | 66.7 | 12 |  |
|  | **80 – 89** | 3 | 33.3 | 2 | 22.2 | 1 | 11.1 | 3 | 33.3 | 9 |  |
|  | **≥ 90** | - | - | - | - | - | - | - | - | 0 |  |
|  | **Total** | 7 | 19.4 | 14 | 38.9 | 3 | 8.3 | 12 | 33.3 | 36 |  |
|  | | | | | | | | | | |  |
| Colorectum | **<60** | 7 | 23.3 | 21 | 70.0 | - | - | 2 | 6.7 | 30 |  |
|  | **60 – 69** | 22 | 11.1 | 135 | 68.2 | 10 | 5.1 | 31 | 15.7 | 198 |  |
|  | **70 – 79** | 62 | 14.3 | 237 | 54.6 | 34 | 7.8 | 101 | 23.3 | 434 |  |
|  | **80 – 89** | 40 | 16.6 | 99 | 41.1 | 29 | 12.0 | 73 | 30.3 | 241 |  |
|  | **≥ 90** | 2 | 16.7 | 4 | 33.3 | 3 | 25.0 | 3 | 25.0 | 12 |  |
|  | **Total** | 133 | 14.5 | 496 | 54.2 | 76 | 8.3 | 210 | 23.0 | 915 |  |
|  | | | | | | | | | | |  |
| Anus | **<60** | - | - | - | - | - | - | - | - | 0 |  |
|  | **60 – 69** | 2 | 100.0 | - | - | - | - | - | - | 2 |  |
|  | **70 – 79** | 1 | 33.3 | 1 | 33.3 | 1 | 33.3 | - | - | 3 |  |
|  | **80 – 89** | - | - | - | - | 2 | 66.7 | 1 | 33.3 | 3 |  |
|  | **≥ 90** | - | - | - | - | - | - | 1 | 100.0 | 1 |  |
|  | **Total** | 3 | 33.3 | 1 | 11.1 | 3 | 33.3 | 2 | 22.2 | 9 |  |
|  | | | | | | | | | | |  |
| Liver | **<60** | - | - | 4 | 80.0 | - | - | 1 | 20.0 | 5 |  |
|  | **60 – 69** | 2 | 4.5 | 33 | 75.0 | 5 | 11.4 | 4 | 9.1 | 44 |  |
|  | **70 – 79** | 8 | 15.4 | 37 | 71.2 | 4 | 7.7 | 3 | 5.8 | 52 |  |
|  | **80 – 89** | 4 | 21.1 | 11 | 57.9 | 2 | 10.5 | 2 | 10.5 | 19 |  |
|  | **≥ 90** | - | - | - | - | - | - | - | - | 0 |  |
|  | **Total** | 14 | 11.7 | 85 | 70.8 | 11 | 9.2 | 10 | 8.3 | 120 |  |
|  | | | | | | | | | | |  |
| Pancreas | **<60** | - | - | 15 | 93.8 | - | - | 1 | 6.3 | 16 |  |
|  | **60 – 69** | 2 | 2.0 | 94 | 92.2 | 3 | 2.9 | 3 | 2.9 | 102 |  |
|  | **70 – 79** | 5 | 6.0 | 73 | 86.9 | 2 | 2.4 | 4 | 4.8 | 84 |  |
|  | **80 – 89** | 1 | 4.3 | 17 | 73.9 | 2 | 8.7 | 3 | 13.0 | 23 |  |
|  | **≥ 90** | - | - | - | - | - | - | 2 | 100.0 | 2 |  |
|  | **Total** | 8 | 3.5 | 199 | 87.7 | 7 | 3.1 | 13 | 5.7 | 227 |  |
| Nose | **<60** | - | - | - | - | 1 | 100.0 | - | - | 1 |  |
|  | **60 – 69** | - | - | - | - | 1 | 100.0 | - | - | 1 |  |
|  | **70 – 79** | - | - | - | - | 1 | 100.0 | - | - | 1 |  |
|  | **80 – 89** | 1 | 25.0 | 1 | 25.0 | 1 | 25.0 | 1 | 25.0 | 4 |  |
|  | **≥ 90** | - | - | - | - | - | - | - | - | 0 |  |
|  | **Total** | 1 | 14.3 | 1 | 14.3 | 4 | 57.1 | 1 | 14.3 | 7 |  |
|  | | | | | | | | | | |  |
| Lungs | **<60** | 1 | 2.4 | 37 | 90.2 | 3 | 7.3 | - | - | 41 |  |
|  | **60 – 69** | 10 | 4.5 | 188 | 85.5 | 9 | 4.1 | 13 | 5.9 | 220 |  |
|  | **70 – 79** | 30 | 9.8 | 230 | 75.4 | 19 | 6.2 | 26 | 8.5 | 305 |  |
|  | **80 – 89** | 13 | 13.3 | 66 | 67.3 | 5 | 5.1 | 14 | 14.3 | 98 |  |
|  | **≥ 90** | - | - | 1 | 50.0 | - | - | 1 | 50.0 | 2 |  |
|  | **Total** | 54 | 8.1 | 522 | 78.4 | 36 | 5.4 | 54 | 8.1 | 666 |  |
|  | | | | | | | | | | |  |
| Breast | **<60** | - | - | - | - | - | - | - | - | 0 |  |
|  | **60 – 69** | - | - | 1 | 100.0 | - | - | - | - | 1 |  |
|  | **70 – 79** | - | - | - | - | 1 | 50.0 | 1 | 50.0 | 2 |  |
|  | **80 – 89** | 2 | 100.0 | - | - | - | - | - | - | 2 |  |
|  | **≥ 90** | - | - | - | - | - | - | - | - | 0 |  |
|  | **Total** | 2 | 40.0 | 1 | 20.0 | 1 | 20.0 | 1 | 20.0 | 5 |  |
|  | | | | | | | | | | |  |
| Testis | **<60** | - | - | - | - | - | - | - | - | 0 |  |
|  | **60 – 69** | - | - | 1 | 50.0 | - | - | 1 | 50.0 | 2 |  |
|  | **70 – 79** | 1 | 100.0 | - | - | - | - | - | - | 1 |  |
|  | **80 – 89** | 1 | 50.0 | 1 | 50.0 | - | - | - | - | 2 |  |
|  | **≥ 90** | - | - | - | - | - | - | - | - | 0 |  |
|  | **Total** | 2 | 40.0 | 2 | 40.0 | - | 0.0 | 1 | 20.0 | 5 |  |
|  | | | | | | | | | | |  |
| Other male genitals | **<60** | - | - | - | - | - | - | - | - | 0 |  |
|  | **60 – 69** | 1 | 25.0 | 1 | 25.0 | 1 | 25.0 | 1 | 25.0 | 4 |  |
|  | **70 – 79** | 7 | 63.6 | 2 | 18.2 | 0 | 0.0 | 2 | 18.2 | 11 |  |
|  | **80 – 89** | 4 | 80.0 | - | - | - | - | 1 | 20.0 | 5 |  |
|  | **≥ 90** | 1 | 100.0 | - | - | - | - | - | - | 1 |  |
|  | **Total** | 13 | 61.9 | 3 | 14.3 | 1 | 4.8 | 4 | 19.0 | 21 |  |
|  | | | | | | | | | | |  |
| Kidney | **<60** | - | - | 6 | 66.7 | 1 | 11.1 | 2 | 22.2 | 9 |  |
|  | **60 – 69** | 11 | 19.3 | 31 | 54.4 | 5 | 8.8 | 10 | 17.5 | 57 |  |
|  | **70 – 79** | 13 | 17.1 | 33 | 43.4 | 8 | 10.5 | 22 | 28.9 | 76 |  |
|  | **80 – 89** | 6 | 27.3 | 8 | 36.4 | 4 | 18.2 | 4 | 18.2 | 22 |  |
|  | **≥ 90** | - | - | - | - | - | - | - | - | 0 |  |
|  | **Total** | 30 | 18.3 | 78 | 47.6 | 18 | 11.0 | 38 | 23.2 | 164 |  |
|  | | | | | | | | | | |  |
| Bladder | **<60** | 1 | 5.9 | 14 | 82.4 | - | - | 2 | 11.8 | 17 |  |
|  | **60 – 69** | 33 | 28.9 | 46 | 40.4 | 13 | 11.4 | 22 | 19.3 | 114 |  |
|  | **70 – 79** | 77 | 30.8 | 79 | 31.6 | 28 | 11.2 | 66 | 26.4 | 250 |  |
|  | **80 – 89** | 57 | 32.8 | 38 | 21.8 | 21 | 12.1 | 58 | 33.3 | 174 |  |
|  | **≥ 90** | 2 | 9.5 | 1 | 4.8 | 5 | 23.8 | 13 | 61.9 | 21 |  |
|  | **Total** | 170 | 29.5 | 178 | 30.9 | 67 | 11.6 | 161 | 28.0 | 576 |  |
|  | | | | | | | | | | |  |
| Melanoma | **<60** | 2 | 33.3 | 3 | 50.0 | 1 | 16.7 | - | - | 6 |  |
|  | **60 – 69** | 15 | 34.9 | 17 | 39.5 | 2 | 4.7 | 9 | 20.9 | 43 |  |
|  | **70 – 79** | 22 | 24.2 | 26 | 28.6 | 8 | 8.8 | 35 | 38.5 | 91 |  |
|  | **80 – 89** | 9 | 17.0 | 7 | 13.2 | 5 | 9.4 | 32 | 60.4 | 53 |  |
|  | **≥ 90** | - | - | 1 | 100.0 | - | - | - | - | 1 |  |
|  | **Total** | 48 | 24.7 | 54 | 27.8 | 16 | 8.2 | 76 | 39.2 | 194 |  |
|  | | | | | | | | | | |  |
| Skin (squamous cell carcinoma) | **<60** | 2 | 50.0 | 2 | 50.0 | - | - | - | - | 4 |  |
|  | **60 – 69** | 19 | 37.3 | 5 | 9.8 | 8 | 15.7 | 19 | 37.3 | 51 |  |
|  | **70 – 79** | 74 | 32.6 | 1 | 0.4 | 29 | 12.8 | 123 | 54.2 | 227 |  |
|  | **80 – 89** | 63 | 28.5 | 1 | 0.5 | 12 | 5.4 | 145 | 65.6 | 221 |  |
|  | **≥ 90** | 6 | 50.0 | - | - | - | - | 6 | 50.0 | 12 |  |
|  | **Total** | 164 | 31.8 | 9 | 1.7 | 49 | 9.5 | 293 | 56.9 | 515 |  |
| Eye | **<60** | - | - | - | - | - | - | - | - | 0 |  |
|  | **60 – 69** | 2 | 50.0 | 1 | 25.0 | - | - | 1 | 25.0 | 4 |  |
|  | **70 – 79** | 1 | 20.0 | - | - | 2 | 40.0 | 2 | 40.0 | 5 |  |
|  | **80 – 89** | - | - | - | - | - | - | 2 | 100.0 | 2 |  |
|  | **≥ 90** | - | - | - | - | - | - | - | - | 0 |  |
|  | **Total** | 3 | 27.3 | 1 | 9.1 | 2 | 18.2 | 5 | 45.5 | 11 |  |
|  | | | | | | | | | | |  |
| Nervous system | **<60** | - | - | 13 | 92.9 | - | - | 1 | 7.1 | 14 |  |
|  | **60 – 69** | 6 | 11.8 | 38 | 74.5 | 4 | 7.8 | 3 | 5.9 | 51 |  |
|  | **70 – 79** | 6 | 14.0 | 22 | 51.2 | 5 | 11.6 | 10 | 23.3 | 43 |  |
|  | **80 – 89** | 1 | 16.7 | 1 | 16.7 | 2 | 33.3 | 2 | 33.3 | 6 |  |
|  | **≥ 90** | - | - | - | - | - | - | - | - | 0 |  |
|  | **Total** | 13 | 11.4 | 74 | 64.9 | 11 | 9.6 | 16 | 14.0 | 114 |  |
|  | | | | | | | | | | |  |
| Thyroid gland | **<60** | 1 | 50.0 | 1 | 50.0 | - | - | - | - | 2 |  |
|  | **60 – 69** | - | - | 4 | 57.1 | 2 | 28.6 | 1 | 14.3 | 7 |  |
|  | **70 – 79** | - | - | 8 | 88.9 | - | - | 1 | 11.1 | 9 |  |
|  | **80 – 89** | - | - | - | - | - | - | 2 | 100.0 | 2 |  |
|  | **≥ 90** | - | - | - | - | - | - | - | - | 0 |  |
|  | **Total** | 1 | 5.0 | 13 | 65.0 | 2 | 10.0 | 4 | 20.0 | 20 |  |
|  | | | | | | | | | | |  |
| Endocrine glands | **<60** | - | - | - | - | - | - | - | - | 0 |  |
|  | **60 – 69** | 3 | 33.3 | - | - | 1 | 11.1 | 5 | 55.6 | 9 |  |
|  | **70 – 79** | 4 | 36.4 | - | - | 1 | 9.1 | 6 | 54.5 | 11 |  |
|  | **80 – 89** | 3 | 50.0 | - | - | - | - | 3 | 50.0 | 6 |  |
|  | **≥ 90** | - | - | - | - | - | - | - | - | 0 |  |
|  | **Total** | 10 | 38.5 | - | - | 2 | 7.7 | 14 | 53.8 | 26 |  |
|  | | | | | | | | | | |  |
| Bone | **<60** | - | - | - | - | - | - | - | - | 0 |  |
|  | **60 – 69** | - | - | 1 | 33.3 | 1 | 33.3 | 1 | 33.3 | 3 |  |
|  | **70 – 79** | 1 | 25.0 | 3 | 75.0 | - | - | - | - | 4 |  |
|  | **80 – 89** | - | - | - | - | - | - | - | - | 0 |  |
|  | **≥ 90** | - | - | - | - | - | - | - | - | 0 |  |
|  | **Total** | 1 | 14.3 | 4 | 57.1 | 1 | 14.3 | 1 | 14.3 | 7 |  |
|  | | | | | | | | | | |  |
| Connective tissue | **<60** | - | - | 1 | 50.0 | 1 | 50.0 | - | - | 2 |  |
|  | **60 – 69** | 2 | 25.0 | 3 | 37.5 | 3 | 37.5 | - | - | 8 |  |
|  | **70 – 79** | 3 | 20.0 | 4 | 26.7 | 4 | 26.7 | 4 | 26.7 | 15 |  |
|  | **80 – 89** | 5 | 41.7 | 2 | 16.7 | 1 | 8.3 | 4 | 33.3 | 12 |  |
|  | **≥ 90** | - | - | - | - | 1 | 33.3 | 2 | 66.7 | 3 |  |
|  | **Total** | 10 | 25.0 | 10 | 25.0 | 10 | 25.0 | 10 | 25.0 | 40 |  |
|  | | | | | | | | | | |  |
| Non-Hodgkin lymphoma | **<60** | - | - | 2 | 100.0 | - | - | - | - | 2 |  |
|  | **60 – 69** | 8 | 11.9 | 44 | 65.7 | 3 | 4.5 | 12 | 17.9 | 67 |  |
|  | **70 – 79** | 17 | 18.9 | 42 | 46.7 | 8 | 8.9 | 23 | 25.6 | 90 |  |
|  | **80 – 89** | 8 | 13.6 | 25 | 42.4 | 8 | 13.6 | 18 | 30.5 | 59 |  |
|  | **≥ 90** | - | - | - | - | 1 | 100.0 | - | - | 1 |  |
|  | **Total** | 33 | 15.1 | 113 | 51.6 | 20 | 9.1 | 53 | 24.2 | 219 |  |
|  | | | | | | | | | | |  |
| Hodgkin lymphoma | **<60** | - | - | - | - | - | - | - | - | 0 |  |
|  | **60 – 69** | - | - | 3 | 75.0 | - | - | 1 | 25.0 | 4 |  |
|  | **70 – 79** | 1 | 33.3 | 2 | 66.7 | - | - | - | - | 3 |  |
|  | **80 – 89** | - | - | 1 | 100.0 | - | - | - | - | 1 |  |
|  | **≥ 90** | - | - | - | - | - | - | - | - | 0 |  |
|  | **Total** | 1 | 12.5 | 6 | 75.0 | 0 | 0.0 | 1 | 12.5 | 8 |  |
|  | | | | | | | | | | |  |
| Multiple myeloma | **<60** | - | - | 3 | 75.0 | - | - | 1 | 25.0 | 4 |  |
|  | **60 – 69** | 3 | 11.1 | 20 | 74.1 | 1 | 3.7 | 3 | 11.1 | 27 |  |
|  | **70 – 79** | 13 | 18.3 | 42 | 59.2 | 1 | 1.4 | 15 | 21.1 | 71 |  |
|  | **80 – 89** | 5 | 14.7 | 22 | 64.7 | 3 | 8.8 | 4 | 11.8 | 34 |  |
|  | **≥ 90** | - | - | - | - | - | - | - | - | 0 |  |
|  | **Total** | 21 | 15.4 | 87 | 64.0 | 5 | 3.7 | 23 | 16.9 | 136 |  |
| Leukemia | **<60** | 1 | 16.7 | 5 | 83.3 | - | - | - | - | 6 |  |
|  | **60 – 69** | 3 | 4.9 | 36 | 59.0 | 12 | 19.7 | 10 | 16.4 | 61 |  |
|  | **70 – 79** | 11 | 10.6 | 57 | 54.8 | 10 | 9.6 | 26 | 25.0 | 104 |  |
|  | **80 – 89** | 5 | 9.8 | 22 | 43.1 | 9 | 17.6 | 15 | 29.4 | 51 |  |
|  | **≥ 90** | 1 | 100.0 | - | - | - | - | - | - | 1 |  |
|  | **Total** | 21 | 9.4 | 120 | 53.8 | 31 | 13.9 | 51 | 22.9 | 223 |  |
|  | | | | | | | | | | |  |
| Cancer of unknown primary | **<60** | 8 | 42.1 | 4 | 21.1 | 7 | 36.8 | - | - | 19 |  |
|  | **60 – 69** | 27 | 26.7 | 12 | 11.9 | 56 | 55.4 | 6 | 5.9 | 101 |  |
|  | **70 – 79** | 33 | 30.3 | 19 | 17.4 | 48 | 44.0 | 9 | 8.3 | 109 |  |
|  | **80 – 89** | 7 | 19.4 | 8 | 22.2 | 13 | 36.1 | 8 | 22.2 | 36 |  |
|  | **≥ 90** | - | - | - | - | 1 | 50.0 | 1 | 50.0 | 2 |  |
|  | **Total** | 75 | 28.1 | 43 | 16.1 | 125 | 46.8 | 24 | 9.0 | 267 |  |
|  | | | | | | | | | | |  |

Abbreviations:

N, %, frequency and percentage;
